# Parent Communication Prompt to Increase Shared Decision-Making: A New Intervention Approach

**DOI:** 10.3389/fped.2018.00060

**Published:** 2018-03-16

**Authors:** Lauren M. Hubner, Heidi M. Feldman, Lynne C. Huffman

**Affiliations:** ^1^Department of Pediatrics, Division of Developmental-Behavioral Pediatrics, Stanford University, Stanford, CA, United States

**Keywords:** shared decision-making, medical decision-making, doctor-patient communication, developmental-behavioral pediatrics, neurodevelopmental disorders

## Abstract

**Objective:**

Shared decision-making (SDM) is the process by which patients, clinicians, and in pediatrics, parents/caregivers, discuss treatment options, communicate available evidence for or against the different options, share preferences and values, and eventually arrive at a joint decision. This study evaluates the use of a novel, universally applicable, SDM intervention, provided to parents, intended to promote engagement and participation with their child’s clinician.

**Methods:**

Two-arm randomized controlled trial comparing the impact of a SDM-focused intervention prompt to a neutral comparison prompt on perception of SDM participation. Participants included English-speaking parents of children (0–17 years) attending one Developmental-Behavioral Pediatric (DBP) clinic and their child’s clinician. Prior to visit start, parents received either the intervention prompt encouraging engagement with the clinician in decision-making, or the comparison prompt reminding them to request a school/work excuse note if needed. After the visit, SDM was assessed by both parents and DBP clinicians. SDM was scored as present if the respondent answered “strongly agree” to all SDM-related items. Logistic regression tested effects of visit, child, parent, clinician characteristics, and intervention group status on parent-reported SDM. Cohen’s kappa assessed alignment between parent and clinician perceptions of SDM.

**Results:**

Of 88 parents screened, 50 (61%) met eligibility criteria and agreed to participate (intervention *n* = 26; comparison *n* = 24). Eligible participants (parents and clinicians) for analysis completed the surveys with no missing data. Overall, SDM was present in 76% of parents and 34% of clinicians. With high rates of parent-reported SDM in both intervention and comparison groups, no main intervention effect was detected. Compared to the comparison group, there was greater alignment between parent and clinician perception of SDM in the intervention group.

**Conclusions:**

Parent and clinician enrollment and data collection with minimal loss suggest that this novel approach is easy to use and could be employed in future outpatient studies exploring SDM. In this clinical setting, both intervention and comparison group parents reported high levels of SDM participation and no main group effect was detected. Further study of this novel parent-directed SDM intervention approach is needed in a larger sample with greater variability in parent-reported SDM to determine its efficacy.

## Introduction

Shared decision-making (SDM) is present when all participants (i.e., patients, family members, and clinicians) share information, communicate preferences, and ultimately arrive at a mutually agreed upon treatment plan (1, 2). In the pursuit of quality healthcare, SDM has been linked to increased patient knowledge, decreased decisional conflict, and adherence to treatment recommendations (3, 4). While generally considered useful in situations of medical complexity (5), consistent use of SDM in practice is lacking, and multiple barriers block its implementation (6).

Shared decision-making is distinctive in the field of pediatrics, where parents often play an important proxy role in treatment decisions. Notably, subsets of pediatric patients, including those with neurodevelopmental disorders or greater functional impairment, are at high risk for lacking SDM (1, 7–9). Clinical tools for patient/family use, such as decision aids, are one way to augment the SDM process (10). However, decision aids typically require high levels of health literacy, are lengthy, and are narrow in focus. SDM training for clinicians is another common approach to increasing SDM adoption (10).

In this study, we sought to construct a novel intervention strategy for increasing SDM. Specifically, we provided parents with a brief, universally applicable (i.e., treatment- and condition-independent) communication prompt, just prior to their child’s clinic visit. This all-purpose, easily accessible intervention prompt encouraged parents to ask questions and engage with their child’s clinician around any number of treatment decisions. This intervention was designed specifically to motivate parents, not clinicians, to serve as the agent of change in the encounter. The setting, a Developmental-Behavioral Pediatrics (DBP) outpatient clinic, was selected for its complex patient population and likelihood of multifaceted treatment discussions.

## Materials and Methods

### Study Design

This study was a two-arm randomized controlled trial intended to assess the impact of an intervention communication prompt compared to a comparison communication prompt provided to parents of children attending a single outpatient DBP clinic, on their perception of SDM participation with their child’s clinician. This study was approved and carried out in accordance with the Stanford University Institutional Review Board and informed written consent was obtained from all participants during enrollment.

### Study Population

The study was performed over a 2-month period in 2016 at Lucile Packard Children’s Hospital, in one DBP clinic. The primary investigator attempted to approach all parents attending clinic during this time period in order to determine their eligibility for enrollment and interest in the study. Parents were eligible for participation if their primary language was English, and their child was <18 years old. Parents were excluded if their child was scheduled for a team evaluation because of the difficulty this would pose in identifying one corresponding clinician. This was a non-probability sample as some children missed their scheduled appointments and others were not screened because the primary investigator was screening another potential participant at the same time. All DBP clinicians were recruited/consented before parent enrollment.

### Study Protocol

Using a recruitment script to ensure consistency, the primary investigator enrolled parents after they registered for their child’s visit. The recruitment script described that the purpose of the study was to understand more about communication during healthcare visits, and outlined general expectations of study participants. After completion of the enrollment and consent procedures, parent participants completed a parent baseline survey and were then randomized sequentially, using a random number generator, into comparison or intervention groups (11). Based on assignment, parents received a packet that included either the comparison or intervention communication prompt and parent follow-up survey. Parents in both groups were instructed to: (1) read the communication prompt before visit start, (2) complete the parent follow-up survey at visit end, and (3) return the follow-up survey to the front desk to receive a $5 gift card. Clinicians completed a clinician follow-up survey at visit end. Parent and clinician follow-up surveys included corresponding items related to perception of SDM participation during the clinic visit. Parent follow-up surveys also assessed impressions of the clinical visit, including satisfaction, trust in the clinician, and empowerment. Parents and clinicians were blind to parent group assignment.

### Variables

#### Independent Variable: Primary

*Intervention group status*: Exposure to the comparison or intervention communication prompt.

Comparison Communication Prompt (designed to be brief, neutral, and not clinically related): “Please have a seat in the waiting area. If you need a work or school excuse note, tell the medical assistant when you and your child are taken back to the clinic room.”

Intervention Communication Prompt (designed to encourage SDM engagement while being brief and clinically related, but general, promoting dialog about any treatment decision arising during the visit): “You know your child best. You can ask the doctor about risks and benefits of suggested treatments. The doctor wants to hear your questions, ideas, and preferences about treatment options.”

#### Dependent Variable: Primary

*Parent-reported SDM Participation (Parent SDM)*: Dichotomous outcome (Parent SDM/Parent Lack of SDM). Outcome based on parent response to the following four items [from the National Survey of Children with Special Healthcare Needs (12)].

To what extent do you agree or disagree with the following statements:

1. The doctor discussed a range of options to consider for my child’s health care or treatment.
2. The doctor encouraged me to ask questions.
3. The doctor made it easy for me to raise concerns.
4. The doctor considered and respected the health care treatment choices I thought would work best for my child.

Based on visit experience, parents were asked to respond “strongly disagree,” “disagree,” “agree,” or “strongly agree” to each item. Parent SDM was achieved when parent participants answered “strongly agree” to all four items. Any other response indicated a lack of Parent SDM.

#### Independent Variables: Secondary

*Visit*: Type (New, Return).
*Child*: gender; age (0–5, 6–17 years); ethnicity (non-Hispanic, Hispanic); race (white, non-white); functional limitation (never, sometimes/usually/always limited by their condition).
*Parent*: education level (>college degree, ≤college degree).
*Clinician*: role (faculty, trainee, nurse practitioner, psychologist); years in practice (0–10, 11–20, >20 years); gender.

#### Dependent Variables: Secondary

*Parent impressions of the clinic visit*: dichotomous outcomes for three domains (satisfaction/lack of satisfaction; trust/lack of trust; empowerment/lack of empowerment). Outcome was based on parent response to each of the three items, respectively.

To what extent do you agree or disagree with the following statements:

1. Satisfaction: “Overall, I am satisfied with the care that my child received.”
2. Trust: “Overall, I trust the knowledge and recommendations of my child’s doctor.”
3. Empowerment/Intent to Follow-Through: “I will be able to follow-through with the treatment plan made for my child today.”

Based on visit experience, parent responded “strongly disagree,” “disagree,” “agree,” or “strongly agree” to each item. Each domain was achieved when parent participants answered “strongly agree” to the item. Any other response indicated a lack in that domain.

*Clinician-reported SDM participation (Clinician SDM)*: dichotomous outcome (Clinician SDM/Clinician lack of SDM). Outcome based on clinician response to the following four items, modeled from the National Survey of Children with Special Healthcare Needs (12). Defined similarly to the above Parent SDM outcome, but from the clinician perspective.

To what extent do you agree or disagree with the following statements:

1. The parent and I discussed a range of options for the child’s health care/treatment.
2. The parent had the opportunity to ask questions about the health care/treatment plan.
3. The parent had the opportunity to raise concerns about the health care/treatment plan.
4. The parent had the opportunity to bring up health care treatment choices that he/she thought would work best for his/her child.

Based on visit experience, clinician responded “strongly disagree,” “disagree,” “agree,” or “strongly agree” to each item. Clinician SDM was achieved when clinician participants answered “strongly agree” to all four items. Any other response indicated a lack of clinician SDM.

### Data Analysis

Chi-square test of independence to assess group differences based on visit/child/parent/clinician characteristics and primary outcome—parent SDM. Chi-square test of independence to assess associations between parent SDM and parent impression of clinic visit (satisfaction, trust, empowerment). Unadjusted logistic regression model tested effects of visit/child/parent/clinician characteristics, and intervention group status, on parent SDM (adjusted model not performed given sample size). Cohen’s kappa assessed the level of agreement between parent and clinician perceptions of SDM during same visit. Statistical significance was *p* < 0.05. Analyses were conducted using IBM SPSS Statistics 22.0 (SPSS Inc. Armonk, NY, USA) (13).

## Results

### Participant Enrollment

During the enrollment period, 108 patients were scheduled in the DBP clinic. 20 were not screened because they either did not attend their scheduled appointment or were missed by the primary investigator because she was screening another potential participant at the same time. Of the 88 parents screened, 34 were excluded: 20 did not meet inclusion criteria (due to inappropriate visit type (i.e., team evaluation) or non-English language), and 14 declined to participate. The remaining 54 parents were randomized to intervention (*n* = 27) or comparison (*n* = 27) groups. One parent in each group was lost to follow-up (i.e., declined to complete the study). An additional two parents in the comparison group were discontinued from the study because of late recognition of inappropriate visit type. All remaining parent participants were included in analysis (intervention *n* = 26, comparison *n* = 24). Of those included in the analysis, all parent and clinician participants completed each survey, with no missing data.

### Descriptive Characteristics

Half of the children were being seen for new visits, half for return. The majority of children were male (64%), 0–5 years old (66%), non-Hispanic ethnicity (64%), non-white race (58%), and described as sometimes/usually/or always limited by their conditions (70%). One-third of parents (34%) had more than a college education. Clinicians were largely female (90%), faculty (60%), with 11–20 years of practice (44%) (Table 1). There were no significant group (i.e., intervention vs. comparison) differences based on any of the descriptive characteristics.

**Table 1:** Descriptive characteristics of the visit, child, parent, and clinician.

	Surveys (total = 50)^a^	
	n	%

Characteristic		
Visit type		
New/initial	25	50
Return/follow-up	25	50
Child gender		
Male	32	64
Female	18	36
Child age		
0–5 years	33	66
6–17 years	17	34
Child ethnicity		
Non-Hispanic	32	64
Hispanic	18	36
Child race		
White	21	42
Non-White	29	58
Child functional limitation		
Never limited	15	30
Sometimes/usually/always limited	35	70
Parent education level		
≤College degree	33	66
>College	17	34
Clinician role		
Faculty attending	30	60
Other (trainee, NP, or psychologist)	20	40
Clinician number of years in practice		
0–10 years	19	38
11–20 years	22	44
>20 years	9	18
Clinician gender		
Male	5	10
Female	45	90

*^a^Information gathered from 50 parent baseline surveys (completed by each of the 50 parent participants) and 50 clinician follow-up surveys (completed by a total of 13 separate clinician participants)*.

### Outcome Measures

#### Parent Perspective

At visit end, when parent responses from both the intervention and comparison groups were combined, 76% of parent participants reported the presence of SDM participation (parent SDM). We observed strong associations between parent SDM and parent impressions of the clinic visit; when parents reported SDM, they almost always reported satisfaction (97.4%), trust (100.0%), and empowerment (97.4%). When parents reported that SDM participation was lacking, far fewer reported satisfaction (58.3%), trust (58.3%), or empowerment (50%). With high rates of parent SDM in both intervention (73.1%) and comparison (79.2%) groups, there were no significant group differences for parent-reported SDM, the primary outcome (Table 2). We also analyzed the data taking a “worst-case scenario” approach: we included the four subjects originally excluded for loss to follow-up or inappropriate visit type, and assumed that they all reported a lack of SDM, and still found no significant group differences for the outcome of parent SDM.

**Table 2:** Bivariate analysis.

	Parent-reported SDM present				
	Comparison group (total *N* = 24)		Intervention group (total *N* = 26)		*p-*Value^a^
	n	%	n	%	
**Total with parent-reported SDM present**	**19**	**79.2**	**19**	**73.1**	**0.614**

**Characteristic**					

Visit type					
New/initial	7	63.6	12	85.7	0.199
Return/follow-up	12	92.3	7	58.3	0.04
Child gender					
Male	14	82.4	12	80	0.865
Female	5	71.4	7	63.6	0.731
Child age					
0–5 years	11	78.6	13	68.4	0.514
6–17 years	8	80	6	85.7	0.759
Child ethnicity					
Non-Hispanic	10	71.4	14	77.8	0.681
Hispanic	9	90	5	62.5	0.159
Child race					
White	9	81.8	7	70	0.525
Non-White	10	76.9	12	75	0.904
Child functional limitation					
Sometimes/usually/always limited	15	78.9	13	81.3	0.865
Never limited	4	80	6	60	0.427
Parent education level					
≤College degree	13	86.7	11	61.1	0.092
>College	6	66.7	8	100	0.036
Clinician role					
Faculty attending	12	70.6	7	53.8	0.346
Other (trainee, NP, or psychologist)	7	100	12	92.3	0.346
Clinician number of years in practice					
0–10 years	6	85.7	8	66.7	0.347
11–20 years	9	81.8	9	81.8	1
>20 years	4	66.7	2	66.7	1
Clinician gender					
Male	2	50	1	100	0.276
Female	17	85	18	72	0.29

*^a^p-Value presented from Chi-square likelihood ratio Fischer’s exact test*.

When the visit was a return visit, parent SDM was significantly lower in the intervention group, compared to comparison. Among parents with more than a college education, parent SDM was significantly higher in the intervention group, compared to comparison. No difference in parent SDM was found based on any of the other descriptive characteristics (Table 2).

In the unadjusted logistic regression model (Table 3), parents had lower odds of SDM if the child’s visit was conducted by a faculty clinician alone [odds ratio (OR) 0.091, *p* = 0.028] compared to visits conducted with a trainee (e.g., resident and fellow), or by another clinician (e.g., nurse practitioner, and psychologist). No other significant differences were detected in the odds of parent SDM based on intervention group status or any other independent variable.

**Table 3:** Logistic regression (unadjusted).

	Parent-reported SDM present	
	OR (95% CI)	p-Value

Characteristic		
Visit type		
New/Initial	Ref	
Return/Follow-up	1.000 (0.273–3.662)	1
Child gender		
Male	Ref	
Female	0.462 (0.123–1.732)	0.252
Child age		
0–5 years	Ref	
6–17 years	1.750 (0.405–7.562)	0.454
Child ethnicity		
Non-Hispanic	Ref	
Hispanic	1.167 (0.297–4.588)	0.825
Child race		
White	Ref	
Non-White	0.982 (0.263–3.662)	0.979
Child functional limitation		
Sometimes/Usually/Always limited	Ref	
Never limited	2.000 (0.515–7.760)	0.316
Parent education level		
≤College degree	0.571 (0.132–2.469)	0.454
>College	Ref	
Clinician role		
Faculty attending	0.091 (0.011–0.775)	0.028
Other (Trainee, NP, or psychologist)	Ref	
Clinician number of years in practice		
0–10 years	1.400 (0.250–7.830)	0.702
11–20 years	2.250 (0.387–13.067)	0.366
>20 years	Ref	
Clinician gender		
Male	0.429 (0.063–2.930)	0.388
Female	Ref	
Intervention group status		
Comparison	Ref	
Intervention	0.714 (0.192–2.653)	0.615

#### Clinician Perspective

At visit end, 34% of all clinicians reported SDM participation (clinician SDM). Clinicians seeing patients whose parents were in the intervention group were more likely to report SDM participation compared to those seeing patients whose parents were in the comparison group (42.3 vs. 25.0%), though this difference did not reach statistical significance [χ^2^ (1) = 1.67, *p* = 0.197].

#### Parent and Clinician Agreement

To assess group effect on level of agreement between parent and clinician perceptions of SDM participation during the same visit, Cohen’s kappa was calculated. In the intervention group, κ = −0.006 (95% CI, −0.322 to 0.310), *p* = 0.973. In the comparison group κ = −0.226 (95% CI, −0.526 to 0.074), *p* = 0.042 (indicating a fair and statistically significant level of disagreement between parents and clinicians regarding perception of SDM participation).

## Discussion

This study represents the initial exploration of a new intervention approach to encouraging SDM. Previous interventions focused on patients have incorporated decision aids that tend to be lengthy and condition/treatment-specific (14), while many SDM-training interventions have focused on clinicians as the target audience (15). In contrast to these previous approaches, we designed a brief SDM intervention that can be universally applied to any treatment decision and provided to parents to motivate them to be an active member of their child’s care team. Overall, 76% of parent participants reported SDM and no main effect of the intervention group was detected. Parent ratings of SDM were positively associated with parent satisfaction, trust in the clinician, and intent to follow-through with the treatment plan (empowerment). In contrast, clinician perception of SDM was relatively low (34%). Assessment of agreement between parent and clinician perceptions of SDM suggested positive intervention effect, with slightly greater alignment of parent and clinician perceptions of SDM in the intervention group.

Recruitment/enrollment was achievable in this subspecialty clinic. As described in the Participant Enrollment sections, of parents randomized, almost all met criteria for analysis (96% intervention, 89% comparison), and surveys were completed fully by parents and clinicians. These results suggest parent and clinician acceptability of this, or a similar, SDM intervention communication prompt, in this outpatient setting.

In this study, parent SDM was found to be quite high. Several factors could account for this finding. Outpatient DBP appointments are typically quite long (~90 min for a new visit and 45–60 min for a return visit). This extended time may allow parents the opportunity to ask questions, consider treatment options for their child, and provide their perspectives; all key components of the SDM process. Similarly, clinicians may utilize this time to explain potential treatments in depth and answer parent questions. Given the high proportion of parents reporting presence of SDM, sufficient variability in parent responses between the groups may not have been adequate to detect an impact of the communication prompt intervention.

Our finding that parent SDM is associated with positive parent perceptions of the clinic visit (e.g., satisfaction) is congruent with previous studies in adult patients (3) and pediatric patients with autism spectrum disorder (16). Because of these associations, in part, SDM has been embraced as an indicator of healthcare quality, and proposed as one promising strategy to enhance healthcare visits.

In contrast to the high overall proportion of parents reporting SDM participation, overall clinician perception of SDM participation was low. Clinicians’ awareness and understanding of the components of SDM in this particular DBP subspecialty group, due to faculty colleagues’ academic interest in this concept, may have contributed to these findings. Clinicians’ knowledge of what truly constitutes a “shared decision” may have caused them to be more self-critical of the clinical interaction than were parents, and therefore less likely to report SDM. This finding encourages the inclusion of a parent-reported SDM participation outcome in future studies, as a focus limited to clinician-report may be misleading. Interestingly, we discerned an apparent, though not statistically significant, effect of the intervention on clinician report of SDM participation (i.e., clinicians were more likely to report presence of SDM during visits with patients whose parents were in the intervention group, compared to the comparison group). This finding suggests that the parent’s receipt of the intervention communication prompt, as opposed to the comparison prompt, may have indeed encouraged parent engagement in the clinical encounter that was noticed by the clinician and reflected in their assessment of whether SDM was present.

Interestingly, in bivariate analysis, parent SDM was significantly lower in the intervention group compared to the comparison group when the visit was a return visit, but no significant difference was seen by group when the visit was a new visit. This difference may be driven in part by visit length and/or the intervention’s impact on parental expectations. While return DBP visits in this clinic are still relatively long (i.e., 45–60 min), they are considerably shorter than a new DBP visit (i.e., ~90 min). Based on their initial experience, parents may come to expect more time with the clinician than allotted for a return visit, resulting in lower parent SDM. Similarly, the intervention communication prompt may prime parents to expect to have the opportunity to discuss treatment options in depth with the clinician, and if this is not realized, reflected in their lower ratings of parent SDM. However, this finding should be interpreted with caution, as there was no main effect of visit type in the regression model.

It is thought-provoking to consider the unexpected finding in the regression model that parents whose child’s visit was conducted by a faculty physician alone reported lower likelihood of SDM compared to those conducted with the addition of a trainee (e.g., resident and fellow), or another clinician (e.g., nurse practitioner, or psychologist). There are multiple potential reasons for this finding, including that when faculty physicians are working with trainees they may be more likely to present to parents all available treatment options in detail as a way of teaching while providing clinical care. Alternatively, compared to another clinician, faculty physicians may be pulled in multiple directions during clinic time (e.g., seeing some patients independently as well as supervising others, answering clinical questions from clinic staff or other colleagues, and so on) and less available to their patients/parents for questions and general SDM engagement. This finding suggests the need for additional research focused on optimal care delivery models (e.g., team/interdisciplinary visits) that support SDM.

Finally, we noted a statistically significant *lack* of agreement between parent and clinician perception of SDM participation in the comparison group only. This lack of agreement about whether or not SDM was present was not particularly surprising, as researchers have documented significant differences between patient and clinician report of whether or not a treatment decision was even discussed during a visit (17). It also highlights the potential difficulty of designing a parent or clinician self-reported outcome variable that captures true engagement in SDM during a clinical encounter, and emphasizes the possible importance of observer-based SDM outcome measures. The fact that the lack of agreement was slightly less, and not statistically significant, in the intervention group, was quite interesting and possibly suggestive of a positive impact of the intervention (i.e., slightly greater alignment of parent and clinician perceptions of SDM participation when parents received the intervention communication prompt).

We recognize that our study has limitations, including small sample size, a single clinical setting, and parent participant exclusion criteria (i.e., primary language not English, child ≥18 years old, team visit), which may limit the generalizability of our findings. Notably, by including only English-speaking parents, our findings may not apply to non-English speakers due to language, and possibly cultural, differences. In addition, the communication prompts were only assessed in one clinical setting, with long time slots allocated for visits, and may have a different impact if used in a clinic with shorter visits. We also acknowledge that we used only one measure of SDM, a self-reported assessment of perceived participation in SDM, which may not accurately reflect the actual presence or absence of SDM during the clinical encounter. In addition, while we tracked the number of parents who described participating in SDM (the outcome encouraged by the intervention prompt), we did not track the number of parents who asked for a work or school excuse note (which was encouraged by the comparison prompt). The communication prompt referred to “the doctor,” instead of a more general term such as “the clinician” or “the healthcare provider.” While this wording was chosen purposefully to ensure the prompt’s readability; as many parents do not discriminate between the “doctor” and other advanced level practitioners, in future studies alternate wording could be considered. Finally, while parents were encouraged to read the communication prompt before visit start, their behavior was not directly verified as a matter of routine. Nonetheless, our study takes a novel approach to SDM intervention and, as such, findings can inform further study design in an effort to enhance SDM participation. In future work, we plan to explore ways to maintain the acceptability of the intervention communication prompt while enhancing its impact on SDM promotion. We also plan to evaluate this intervention in other pediatric subspecialty clinics, especially those where we would anticipate greater variability in parent-reported SDM. Finally, a future study could attempt to videotape the clinical interactions, in order to directly measure the degree of SDM present.

## Conclusion

A brief, easy to understand, and adaptable clinical tool that can effectively motivate parent engagement in SDM around a multitude of treatment decisions is a needed and novel approach to SDM intervention. This study was a first step in designing such a tool, and assessing its use in one outpatient subspecialty clinic. While we did not demonstrate an overall intervention effect, as a general observation we noted ease of participant enrollment and complete collection of participant survey data, which is suggestive of the intervention’s acceptability to parents and clinicians, and a positive indicator for future studies. Continued work is needed in order to enhance the intervention’s impact on positive perceptions of SDM participation.

## Ethics Statement

This study was carried out in accordance with the recommendations of the Stanford University Institutional Review Board with written informed consent from all subjects. All subjects gave written informed consent in accordance with the Declaration of Helsinki. The protocol was approved by the Stanford University Institutional Review Board.

## Author Contributions

All authors made substantial contributions to either the design of the work, or acquisition, analysis, or interpretation of data for the work, drafted the work or critically revised it, approve the final version to be published, and agree to be accountable for all aspects of the work (LMH, HF, and LCH).

## Conflict of Interest Statement

The authors declare that the research was conducted in the absence of any commercial or financial relationships that could be construed as a potential conflict of interest.

## Abbreviations

SDM, shared decision-making; DBP, developmental-behavioral pediatric.
